# Deciphering the Shared Mechanisms Underlying the Effects of Osthole on the Inflammation–Cancer Axis: An Integrative Network Pharmacology and Molecular Dynamics Study

**DOI:** 10.3390/cimb48050518

**Published:** 2026-05-15

**Authors:** Peng Tang, Jing Yang, Haoyi Wang, Meiqi Zhang, Miao Tian, Yuqin Zhao, Ming Liu, Rui Wang

**Affiliations:** 1 College of Pharmacy, Heilongjiang University of Chinese Medicine, Harbin 150040, China; 17785423768@163.com (P.T.); wanghaoyi0329@163.com (H.W.); 16645410412@163.com (M.Z.); creatora@163.com (M.T.); 18746747325@163.com (M.L.); 2 College of Basic Medical Science, Heilongjiang University of Chinese Medicine, Harbin 150040, China; wyacutedx@163.com (J.Y.); zhaoyuqinyr@163.com (Y.Z.)

**Keywords:** Osthole, inflammation–cancer axis, network pharmacology, molecular dynamics simulation, multi-target mechanism, PI3K/Akt signaling, NF-κB signaling, TGF-β/Smad pathway, tumor microenvironment

## Abstract

The persistence of an immunosuppressive microenvironment remains a formidable challenge for cancer immunotherapy, particularly in tumors with immune-excluded or immune-desert phenotypes. Increasing evidence indicates that chronic inflammation and tumor progression are intrinsically linked through shared signaling hubs, including NF-κB and PI3K/Akt. Osthole, a natural coumarin compound, has been reported to exhibit both potent anti-inflammatory and antitumor activities; however, whether these effects reflect a coordinated regulation of the inflammation–cancer axis remains unclear. In this study, we deployed an integrative framework founded on network pharmacology, molecular docking, and rigorous molecular dynamics simulations, complemented by literature-based evidence synthesis, to computationally explore the potential mechanisms underlying Osthole’s dual activities. Our analysis revealed that Osthole’s predicted targets are significantly enriched in signaling pathways bridging inflammatory and oncogenic processes, most notably the PI3K/Akt, NF-κB, and TGF-β/Smad pathways. Crucially, MD simulations provided supportive computational evidence, suggesting that Osthole forms stable, energetically favorable complexes with core protein hubs (AKT1, RELA, and TGFB1) under the simulated conditions. Evidence from representative inflammatory and tumor models supports the biological plausibility of these predictions, including suppression of pro-inflammatory signaling, mitigation of maladaptive tissue remodeling, and induction of apoptosis. Furthermore, in hepatocellular carcinoma models, Osthole-mediated apoptosis appeared linked to HMGB1-related inflammatory signaling, highlighting its potential to modulate the local immune niche. Collectively, this convergence of systems-level predictions and dynamic structural evidence identifies Osthole as a promising multi-target candidate for the coordinated regulation of inflammation-associated tumor progression, providing a robust rationale for further experimental validation.

## 1. Introduction

Tumor immunotherapy, particularly PD-1 and PD-L1 inhibitors, has achieved breakthrough clinical success across multiple malignancies [1,2,3]. However, overall response rates to immune checkpoint inhibitors (ICIs) remain modest in most solid tumors, where primary or acquired resistance is pervasive [4,5]. Mounting evidence suggests that persistent chronic inflammation and the resulting TME fundamentally restrict antitumor immune responses and blunt immunotherapeutic efficacy [6,7,8,9,10]. Within this context, inflammatory networks continually drive the recruitment and expansion of myeloid-derived suppressor cells (MDSCs), M2-polarized TAMs, and Tregs, forging a robust barrier to immune evasion [11,12,13]. Notably, this inflammation-sustained pro-tumor niche is not a passive backdrop, but a dynamic ecosystem actively driving tumor cell survival and evolution. Consequently, the limited efficacy of immunotherapy in solid tumors stems not merely from intrinsic tumor cell resistance, but from a continuously reinforcing pathological feedback loop among pro-inflammatory signaling, immunosuppression, and malignant phenotypes. Therefore, dismantling this feedback loop via the inflammation–cancer axis, thereby synchronously targeting inflammatory drivers and tumor progression, represents a critical priority in contemporary oncology research.

Inflammation and cancer are not isolated pathological events; rather, they exist along a continuous spectrum characterized by highly coupled molecular networks, microenvironmental shifts, and biological behaviors. Extensive research confirms that chronic inflammation not only shapes the TME during tumor initiation but also continuously fuels proliferation, angiogenesis, invasion, metastasis, and therapeutic resistance [14]. Prolonged inflammatory stimulation induces genomic instability and extracellular matrix remodeling, creating a permissive environment for malignant transformation [15,16]. Once established, tumors secrete additional inflammatory mediators, establishing a positive feedback loop between inflammation and cancer [17,18]. Crucially, signaling pathways such as NF-κB, PI3K/Akt, and TGF-β/Smad exhibit extensive crosstalk in amplifying inflammation, driving tissue remodeling, and sustaining malignant behaviors. This suggests that inflammation and cancer inherently share a common set of core therapeutic targets [19,20,21,22]. Consequently, single-target interventions are rarely sufficient to dismantle the complex molecular networks underlying inflammation-driven tumors. Identifying multi-target agents capable of simultaneously regulating cell fate and halting tumor progression presents a promising translational strategy for the systemic treatment of complex malignancies.

Due to their structural diversity and multi-target profiles, natural products offer distinct advantages in managing complex diseases [23,24,25]. Unlike traditional single-target drugs, bioactive natural small molecules are well-suited for systemic intervention across the intertwined networks of inflammation, cellular stress, and tissue remodeling.

Osthole, a naturally occurring coumarin derivative, has garnered significant interest in its broad pharmacological properties [26,27]. Previous studies indicate that Osthole exhibits dual capacity to mitigate inflammatory injury and tissue remodeling while simultaneously inducing tumor cell apoptosis across various experimental models [28,29,30]. However, most existing research evaluates these effects in isolated disease contexts. It remains unclear whether a shared molecular basis links Osthole’s anti-inflammatory and antitumor activities, and whether these effects are co-regulated through common hub targets and convergent signaling pathways. Whether the dual pharmacological activities of Osthole represent parallel, isolated phenomena or reflect a unified intervention against a shared inflammation–cancer network remains a critical, unanswered question.

Advances in computational pharmacology, particularly network pharmacology, have established a new paradigm for delineating the multi-target and multi-pathway mechanisms of natural products. Integrating systems-level network analysis with structural molecular docking enables the precise identification of convergent nodes targeted by active molecules across distinct pathological states. Furthermore, molecular dynamics (MD) simulations can assess the dynamic stability of predicted ligand–target interactions, providing physicochemical support for mechanistic hypotheses [31,32,33]. Building on this framework, the present study targets the inflammation–cancer axis by integrating network pharmacology, molecular docking, MD simulations, and literature mining to systematically decode the shared targets and convergent pathways of Osthole in inflammation and cancer. Our objective is not merely to confirm Osthole’s dual activities, but to provide deep structural evidence of its synergistic regulatory potential within the shared molecular network. Ultimately, this study aims to elucidate the systemic, microscopic mechanisms by which Osthole intervenes in the inflammation–cancer axis, offering a robust theoretical foundation for the repurpose and development of natural products in complex, remodeling-driven diseases.

## 2. Materials and Methods

### 2.1. Osthole Target Prediction and Disease-Related Gene Collection

Potential targets of Osthole were identified using three complementary approaches. First, the Traditional Chinese Medicine Systems Pharmacology Database and Analysis Platform (TCMSP; https://www.tcmsp-e.com/tcmsp.php, accessed on 24 November 2025) was queried for “Cnidii Fructus.” All protein targets associated with its known active ingredient, “Osthole,” were retrieved. Second, reverse pharmacophore matching was performed using the PharmMapper server (http://www.lilab-ecust.cn/pharmmapper/, accessed on 24 November 2025). The three-dimensional structure of Osthole in MOL2 format, obtained from the PubChem database, was submitted with the search restricted to human protein targets, and only targets with a normalized fit score greater than 0.9 were retained to reduce low-confidence predictions. Third, the SwissTargetPrediction database (http://www.swisstargetprediction.ch/, accessed on 24 November 2025) was used to predict targets based on the canonical SMILES notation of Osthole, which was also sourced from PubChem. Because these three platforms use different algorithms and scoring systems, their outputs were cross-checked rather than merged without filtering. To reduce source-specific false positives, only targets supported by at least two independent databases were retained in the final Osthole candidate target set. Targets from eligible sources were standardized to official human gene symbols using UniProt annotation, restricted to Homo sapiens where applicable, merged, and deduplicated. Thus, the final Osthole target library was considered a curated computational candidate set rather than a list of experimentally validated targets.

Disease-related genes were collected from three primary databases: GeneCards (https://www.genecards.org/, accessed on 26 November 2025), the Online Mendelian Inheritance in Man (OMIM; https://www.omim.org/, accessed on 26 November 2025), and the Therapeutic Target Database (TTD; https://db.idrblab.net/ttd/, accessed on 26 November 2025). For each disease of interest, its standard English name was used as a keyword for parallel searches across these databases. To ensure gene-disease relevance, a filtering threshold was applied within GeneCards, where only genes with a “Relevance score” above the median score for all genes associated with that specific disease were selected. This disease-specific median threshold was used as an adaptive filter because GeneCards score distributions and the number of returned genes vary across diseases; a single fixed cutoff could be overly permissive for well-studied diseases or overly restrictive for less extensively studied conditions. All known disease-associated genes listed in the OMIM and TTD databases were included. The search results from the three databases were then merged and deduplicated to build a distinct target gene library for each disease. Representative inflammatory and tumor-related disease models were retained not to provide exhaustive disease coverage, but to capture biologically distinct yet mechanistically connected contexts across the inflammation–cancer axis. This design allowed us to evaluate whether candidate Osthole-associated targets repeatedly converged on a limited set of shared signaling modules despite differences in tissue origin, pathological trigger, and disease outcome.

### 2.2. Target Intersection Analysis

To identify shared targets between Osthole and each disease, the Osthole Potential Target library was intersected with the respective target library of each disease. All intersection analyses were conducted in the R language environment (version 4.5.1), using the Venn package to perform the set operations and generate the corresponding Venn diagrams.

### 2.3. Construction and Visualization of PPI Networks

The shared targets identified across all studied diseases and Osthole were pooled to form a total target gene set. This gene list was submitted to the STRING database (https://string-db.org/, accessed on 28 November 2025), with the organism limited to Homo sapiens. A medium confidence threshold (minimum required interaction score = 0.400) was applied to filter the interactions, and isolated nodes were excluded from the resulting network. This threshold was selected because the present study focused on an exploratory, systems-level analysis of a natural compound with potential multi-target activity. Using a medium-confidence cutoff helped preserve network connectivity across targets derived from multiple disease contexts, while still applying the standard STRING filtering framework. The retrieved protein–protein interaction data, provided in TSV format, were then imported into Cytoscape software (version 3.9.1) for subsequent network visualization and topological analysis.

### 2.4. Screening of Core Targets

The core hub genes within the PPI network were identified using the cytoHubba plugin in Cytoscape. Node centrality was evaluated with two independent algorithms: MCC and Degree. Genes ranked in the top 20 by both methods were selected as the high-confidence core target set, representing key mediators through which Osthole may act on the inflammation–cancer axis, and were subjected to further analysis.

### 2.5. Gene Ontology and KEGG Pathway Enrichment Analyses

To characterize the biological roles of the shared targets between Osthole and all investigated diseases, functional enrichment analysis was conducted. The R package clusterProfiler (version 4.5.1) was employed for Gene Ontology and Kyoto Encyclopedia of Genes and Genomes pathway enrichment analyses. Input genes were provided in Entrez ID format. The GO analysis covered three categories: Biological Process, Cellular Component, and Molecular Function. Statistically significant terms were defined as those with an adjusted *p* value < 0.05 after false discovery rate correction. For result interpretation, the top 10 most significant GO terms and the top 30 enriched KEGG pathways were selected for visualization. The enrichplot package was used to generate bar charts and bubble plots, effectively illustrating the enrichment outcomes.

### 2.6. Molecular Docking Analysis

The three-dimensional crystal structure of Osthole was retrieved in SDF format from the PubChem database (https://pubchem.ncbi.nlm.nih.gov/, accessed on 22 January 2026). The structure was energy-minimized using ChemBio3D Ultra 14.0 and converted to MOL2 format. AutoDockTools 1.5.7 was then employed to add Gasteiger charges, assign atom types, and export the ligand in PDBQT format for docking. Protein IDs of core targets were obtained from UniProt (https://www.uniprot.org/, accessed on 22 January 2026). Corresponding crystal structures were downloaded from the RCSB PDB database (https://www.rcsb.org/, accessed on 22 January 2026) using the respective sequence identifiers. Each protein structure was prepared in PyMOL 3.1.2 by removing native ligands, water molecules, and non-essential ions, adding hydrogen atoms, and saving them in PDBQT format.

Molecular docking was performed using AutoDock Vina 1.1.2 with its default stochastic global optimization and local search procedure. The grid center coordinates and grid box dimensions used for each receptor are provided in Supplementary Table S1. Each docking run was repeated 10 times independently, and the conformation with the lowest calculated binding free energy (ΔG, in kcal/mol) was selected as the optimal binding mode. Docking energies below −5.0 kcal/mol were used as an exploratory threshold to retain possible ligand–target binding poses and were not interpreted as evidence of strong or specific binding. Redocking validation was performed for protein structures containing suitable co-crystallized small-molecule ligands. The native ligand was extracted and re-docked into the crystallographic binding pocket using the same receptor preparation, grid definition, and docking workflow as those used for Osthole. The re-docked pose was superimposed onto the experimental ligand pose, and the heavy-atom root mean square deviation (RMSD) was calculated. RMSD values below 2.0 Å were considered acceptable for reproducing the crystallographic binding mode. Reference-inhibitor docking was performed to provide a relative benchmark for the docking scores of Osthole. For each target, one representative inhibitor reported in the literature or commonly used as a pathway-relevant inhibitor was selected. All reference inhibitors were docked using the same receptor preparation, grid definition, and docking parameters as those used for Osthole.

The three-dimensional binding poses of Osthole within each target protein were visualized using PyMOL. Two-dimensional interaction diagrams were generated with Discovery Studio 4.5 Client to annotate key molecular interactions, including hydrogen bonds and hydrophobic contacts.

### 2.7. Molecular Dynamics Simulation

Molecular dynamics simulations of the protein-ligand complexes were performed in GROMACS 2025.2. The AMBER99SB-ILDN and GAFF force fields were applied to proteins and ligands, respectively, and AM1-BCC partial atomic charges were assigned to Osthole. Each complex was solvated with TIP3P water molecules within a periodic simulation box, maintaining a minimum distance of 1.0 nm between the solute and the box boundaries. Counterions were added to neutralize the systems. Initial energy minimization was conducted using the steepest descent algorithm until the maximum force fell below 100.0 kJ/mol/nm, followed by a second minimization step using the conjugate gradient method. The systems were then equilibrated into two sequential 2 ns phases (1,000,000 steps each) under NVT and NPT ensembles. A constant temperature of 300 K and pressure of 1.0 bar were maintained throughout equilibration and subsequent runs. Following the removal of position restraints, 100 ns production MD simulations were executed for each system with a 2-fs time step. Long-range electrostatics were computed via the Particle Mesh Ewald method. Cutoff distances for both short-range electrostatic and van der Waals interactions were set to 1.0 nm. The LINCS algorithm was utilized to constrain all bonds involving hydrogen atoms. Trajectory analysis, including RMSD, root mean square fluctuation (RMSF), radius of gyration (Rg) and solvent accessible surface area (SASA), was performed using standard GROMACS utilities. Binding free energies of the complexes were calculated using gmx_MMPBSA (v1.6.1; [34]). It should be noted that MM/PBSA-derived binding energies are approximate estimates dominated by enthalpic contributions. Because entropic effects were not explicitly included, these values were used primarily to compare relative binding tendencies among different complexes rather than to represent rigorous absolute thermodynamic binding free energies.

### 2.8. Literature-Based Evidence Synthesis

To contextualize the computational predictions within published biological evidence, a literature-based evidence synthesis was conducted. PubMed, Web of Science, and X-MOL were searched using combinations of “Osthole” with core targets, enriched signaling pathways, and representative disease models identified in the computational analyses. Example search terms included (“Osthole”) AND (“AKT1” OR “PI3K/Akt signaling pathway”) and (“Osthole”) AND (“allergic asthma” OR “hepatocellular carcinoma”). Retrieved studies were used to examine whether published experimental findings were biologically consistent with the signaling patterns suggested by network pharmacology, molecular docking, and molecular dynamics simulations. This step was not designed as a systematic review or meta-analysis. Instead, it was used to provide mechanistic context and assess whether the computationally prioritized targets and pathways were supported by previously reported experimental observations.

## 3. Results

### 3.1. Overall Construction of the Osthole–Disease Interaction Network and Screening of Core Targets

**Figure 1:**
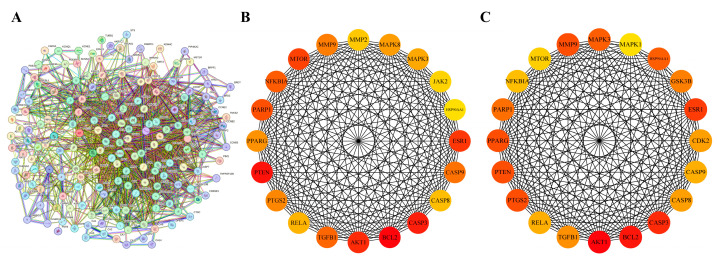
Identification of hub targets in the Osthole-associated PPI network: (**A**) PPI network of the pooled shared targets between Osthole and the selected disease models, generated and exported directly from STRING. Nodes represent target proteins, and edges represent predicted or known protein–protein interactions. (**B**) Top 20 hub genes ranked by MCC using the cytoHubba plugin in Cytoscape. (**C**) Top 20 hub genes ranked by Degree using the cytoHubba plugin in Cytoscape. Genes overlapping between the MCC and Degree rankings were retained as core targets for subsequent analyses.

The shared targets between Osthole and each of the nine diseases were first identified individually and then pooled. After duplicate removal, this yielded 176 unique shared targets pooled shared targets across all nine disease contexts. The constructed PPI network displayed clear biological organization, with key topological metrics as follows: number of nodes: 176; number of edges: 1874; average node degree: 21.3; average local clustering coefficient: 0.58. The network showed significant enrichment of interactions compared with a random model (expected number of edges: 776; PPI enrichment *p*-value: <1.0 × 10^−16^), indicating its non-random, modular structure relevant to biological function (Figure 1A). To identify the most influential proteins, we evaluated network hubs using two complementary centrality algorithms. The MCC algorithm pinpoints proteins that bridge multiple functional modules, while Degree centrality measures the number of direct interactions. Both methods consistently highlighted a core set of proteins (Figure 1B,C). Notably, 17 genes ranked among the top 20 hubs in both analyses, including well-established regulators of inflammation and cancer such as AKT1, RELA, CASP3, CASP8, CASP9, PTGS2, and TGFB1. The overlap indicates that the pharmacological effects of Osthole are more likely to converge on a limited group of central signaling hubs than to reflect a broad set of unrelated targets. These overlapping hub genes were therefore considered the global core targets of the present study. In contrast, the docking targets selected in the individual disease sections were used as context-specific validation nodes to illustrate how the shared signaling framework of Osthole may manifest in different pathological settings.

### 3.2. Biological Function and Pathway Enrichment Analysis of the Core Target Set

**Figure 2:**
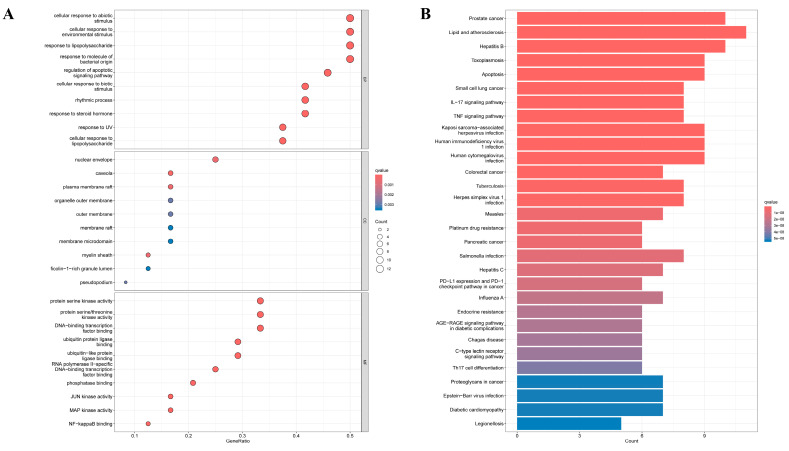
GO and KEGG enrichment analyses of the core targets: (**A**) GO enrichment analysis of the core targets, including BP, CC, and MF categories. (**B**) KEGG pathway enrichment analysis of the core targets. The enrichment analyses were performed using clusterProfiler, and significant terms/pathways were ranked according to adjusted *p* values. Color represents enrichment significance, and dot size represents gene count where applicable.

To elucidate the biological implications of the core Osthole-targeted gene set, we performed GO and KEGG pathway enrichment analyses. GO analysis offered functional insight, revealing that the core genes were significantly enriched in key biological processes including response to lipopolysaccharide, response to molecule of bacterial origin, and the critical regulation of apoptotic signaling pathway, which collectively underscore Osthole’s potential to modulate infectious inflammation and programmed cell death (Figure 2A). Notably, NF-κB binding emerged as one of the most enriched molecular functions, suggesting a possible association between Osthole-related targets and NF-κB-associated regulatory processes. Concurrently, enrichment in protein serine/threonine kinase activity, MAP kinase activity, and JUN kinase activity aligned with the PI3K/Akt and MAPK signaling pathways identified in our KEGG analysis. Cellular component terms such as nuclear envelopes, plasma membrane rafts, and organelle outer membrane further suggested that Osthole’s influence may initiate at membrane signaling hubs and ultimately affect nuclear gene transcription events.

KEGG pathway enrichment analysis further delineated the coordinated molecular network through which Osthole may act (Figure 2B). The top 30 enriched pathways were grouped into four functional categories based on biological relevance and statistical significance (*p.adjust* < 0.0001) (Table 1). These results place the core targets of Osthole within a highly interconnected signaling network that governs both inflammation and cancer-related processes. Key inflammatory drivers such as the IL-17 and TNF signaling pathways were prominently enriched, along with central survival and metabolic hubs like the PI3K/Akt signaling pathway. Importantly, the NF-κB signaling pathway, a well-established bridge linking chronic inflammation to malignancy, was also significantly enriched. The transcription factor RELA, a shared component across several of these pathways, may serve as an integrative node that coordinates upstream signals to activate downstream pro-tumorigenic programs. Furthermore, enrichment of the Apoptosis pathway is consistent with the cytotoxic effects reported for Osthole in tumor models, while enrichment of the PD-L1 expression and PD-1 checkpoint pathway in cancer suggests possible relevance to immune-regulatory processes within the tumor microenvironment. However, whether Osthole directly modulates these processes remains to be experimentally determined. Viewed together, the GO and KEGG results suggest that Osthole may act on the inflammation–cancer axis through a coordinated network involving inflammatory signaling, signal integration, cell-fate control, and immune-related pathways. This enrichment pattern provided a functional context for the subsequent disease-specific analyses.

### 3.3. Molecular Docking Analysis of Osthole with Representative Global and Context-Specific Targets

To provide structural support for the predicted target-pathway associations, molecular docking was conducted using representative targets at two complementary levels. At the global level, selected proteins from the pooled cross-disease core target set were docked with Osthole to assess the structural plausibility of the principal signaling modules identified by network pharmacology. At the disease-specific level, one representative target was selected within each pathological context to reflect the local mechanistic relevance of Osthole–target interactions in that model. Detailed docking parameters, including grid center coordinates and grid box dimensions for each receptor, are provided in Supplementary Table S1. Redocking validation was performed for targets with suitable co-crystallized small-molecule ligands in the original PDB structures. The native ligands used for redocking and the corresponding RMSD values are summarized in Supplementary Table S2. RMSD values below 2.0 Å were considered acceptable for reproducing the crystallographic binding pose. For targets lacking suitable co-crystallized small-molecule ligands, redocking validation was not applicable.

The docking results are summarized in Table 2. Osthole showed favorable predicted docking scores across all selected targets, with binding energies ranging from −6.2 to −8.6 kcal/mol. Among the selected targets, the lowest predicted binding energy was observed for PTGS2, followed by AKT1, PPARG, MTOR, TGFB1, ESR1, and CASP9. When docked under the same conditions, selected reference comparators showed binding energies in a similar range. Osthole yielded comparable or lower predicted binding energies for several representative targets, including AKT1, CASP8, CASP9, ESR1, HSP90AA1, MMP9, MTOR, NFKBIA, PTGS2, RELA, and TGFB1. These comparisons provide a reference frame for interpreting the relative docking performance of Osthole across the selected targets.

### 3.4. From Inflammatory Persistence to Tissue Remodeling

#### 3.4.1. Airway Inflammation at the Onset of Remodeling

**Figure 3:**
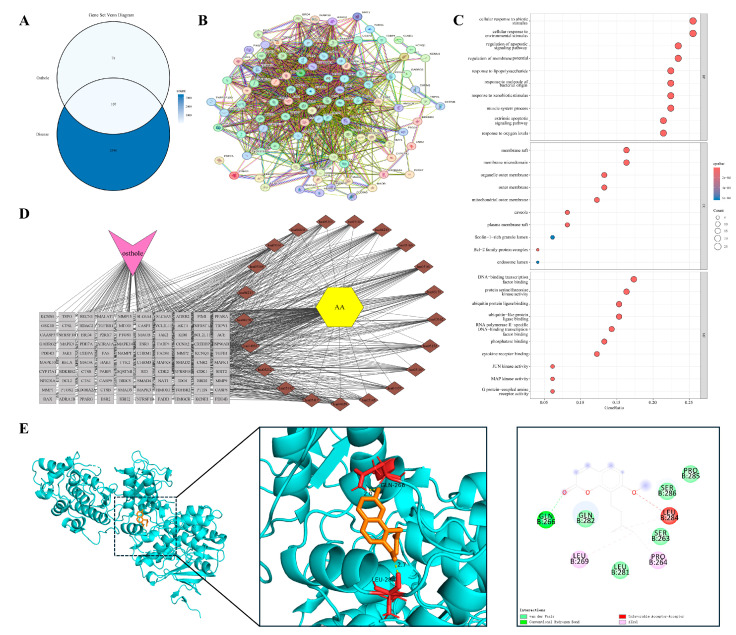
Network pharmacology and molecular docking analysis of Osthole in allergic asthma: (**A**) Venn diagram showing the intersection between predicted Osthole targets and AA-associated genes; 107 overlapping targets were retained for further analysis. (**B**) PPI network of the overlapping targets generated using STRING and visualized in Cytoscape. (**C**) GO enrichment analysis of the overlapping targets, including BP, CC, and MF categories. Dot size represents gene count, and dot color represents enrichment significance. (**D**) Drug–target–disease–pathway network showing predicted associations among Osthole, overlapping targets, enriched KEGG pathways, and AA. (**E**) Docking analysis of Osthole with MAPK3 (PDB ID: 2ZOQ), including the overall 3D binding pose, magnified binding pocket, and 2D ligand–residue interaction diagram.

The progression from persistent inflammation to pathological tissue remodeling provides a framework for examining the inflammation–cancer axis. We used allergic asthma as a representative model of this continuum. In contrast to the traditional focus on acute bronchospasm, chronic asthma is characterized by early airway remodeling driven by a persistent inflammatory microenvironment. Overlap analysis identified 107 shared targets between Osthole and allergic asthma (Figure 3A). Protein–protein interaction (PPI) analysis based on STRING suggested that these targets were organized into an interconnected network rather than appearing as isolated proteins or a single functional module. Representative nodes clustered into several functional groups, including inflammatory signaling (RELA, TNFRSF1A, NFKBIA), kinase cascades (AKT1, MAPK1/3), cell-fate regulation (CASP3, BCL2), and matrix remodeling (MMP9, TGFB1) (Figure 3B). This modular organization recurred in subsequent inflammation-remodeling models and provided a framework for disease-specific comparisons. Functional enrichment analysis (Figure 3C,D) indicated that these targets were enriched in cytokine receptor binding, regulation of apoptotic signaling, and serine/threonine kinase activities, particularly the MAPK pathway, rather than being limited to basal immune functions. These results suggest that the Osthole-associated target set in asthma may be linked to inflammatory signaling, cell-fate control, and remodeling-related pathways, indicating a broader predicted relevance beyond symptomatic airway relaxation. Within this network, MAPK3 was selected as a representative docking target because of its role in transmitting upstream inflammatory stimuli to downstream transcriptional programs. Molecular docking predicted a favorable binding pose of Osthole within the MAPK3 pocket, with a calculated binding energy of −6.9 kcal/mol and key contacts involving GLN-266 and LEU-284 (Table 2 and Figure 3E).

#### 3.4.2. Barrier Failure and Self-Sustaining Cutaneous Inflammation

**Figure 4:**
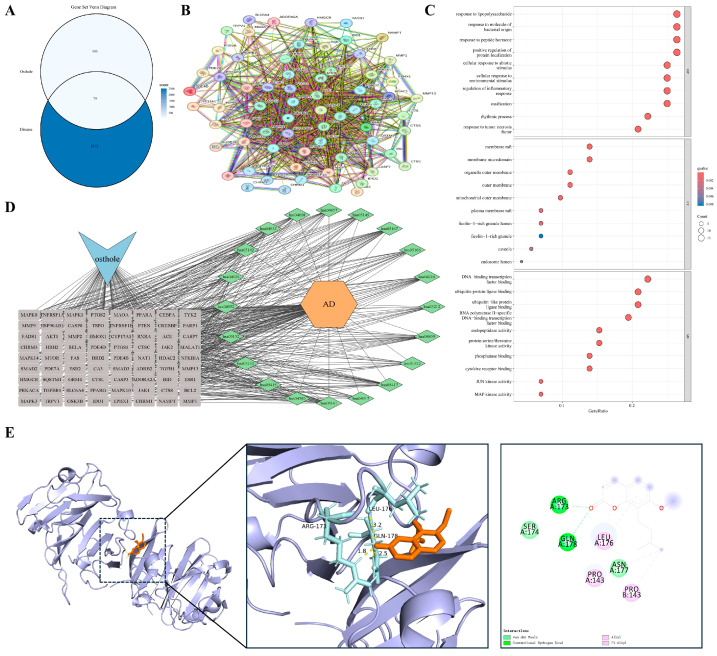
Network pharmacology and molecular docking analysis of Osthole in AD: (**A**) Venn diagram showing the intersection between predicted Osthole targets and AD-associated genes; 79 overlapping targets were retained for further analysis. (**B**) PPI network of the overlapping targets generated using STRING and visualized in Cytoscape. (**C**) GO enrichment analysis of the overlapping targets, including BP, CC, and MF categories. Dot size represents gene count, and dot color represents enrichment significance. (**D**) Drug–target–disease–pathway network showing predicted associations among Osthole, overlapping targets, enriched KEGG pathways, and AD. (**E**) Docking analysis of Osthole with MMP9 (PDB ID: 1ITV), including the overall 3D binding pose, magnified binding pocket, and 2D ligand–residue interaction diagram.

Paralleling the airway mucosal dynamics, impaired cutaneous epithelial barrier function provides another model for studying self-sustaining chronic inflammation. Atopic dermatitis (AD) is characterized by compromised barrier integrity and polarized immune responses that create a reciprocal feed-forward cycle, potentially contributing to long-term tissue architectural changes. We identified 79 shared targets between Osthole and AD-related genes (Figure 4A). Consistent with the modular pattern observed in the asthma model, PPI analysis suggested an interconnected network rather than a set of isolated proteins. Representative nodes associated with inflammatory amplification and barrier remodeling included RELA, TNFRSF1A, PTGS2, MMP9, and TGFB1 (Figure 4B).

Enrichment analysis (Figure 4C,D) indicated that these candidates were concentrated in the tumor necrosis factor (TNF) signaling pathway, regulation of inflammatory responses, and serine/threonine kinase activities. Enrichment of responses to lipopolysaccharide and molecules of bacterial origin was consistent with the barrier-disrupted nature of AD, suggesting that the Osthole-associated target set may be linked to both inflammatory signaling and microbe-associated stimulation. This profile places the AD-related targets at the intersection of barrier failure, microbial exposure, and sustained inflammation. MMP9 was selected as the representative docking target because of its role in tissue remodeling and epithelial barrier degradation. Docking predicted a favorable Osthole-binding pose with a calculated binding energy of −7.1 kcal/mol and key contacts with ARG-173, GLN-178, and LEU-176 (Table 2 and Figure 4E).

#### 3.4.3. Chronic Synovitis with Tumor-like Tissue Invasion

**Figure 5:**
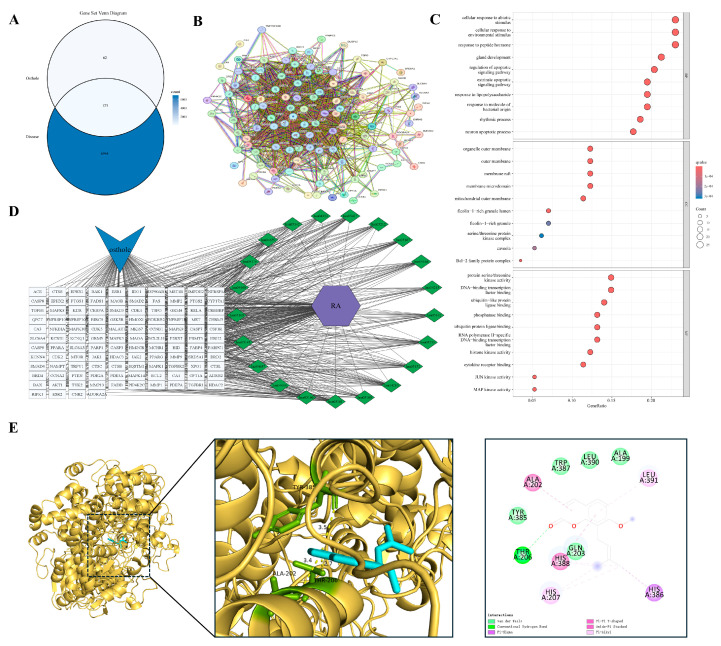
Network pharmacology and molecular docking analysis of Osthole in RA: (**A**) Venn diagram showing the intersection between predicted Osthole targets and RA-associated genes; 123 overlapping targets were retained for further analysis. (**B**) PPI network of the overlapping targets generated using STRING and visualized in Cytoscape. (**C**) GO enrichment analysis of the overlapping targets, including BP, CC, and MF categories. Dot size represents gene count, and dot color represents enrichment significance. (**D**) Drug–target–disease–pathway network showing predicted associations among Osthole, overlapping targets, enriched KEGG pathways, and RA. (**E**) Docking analysis of Osthole with PTGS2 (PDB ID: 5F19), including the overall 3D binding pose, magnified binding pocket, and 2D ligand–residue interaction diagram.

When chronic inflammation extends beyond localized barrier interfaces, its pathological manifestations often evolve into invasive tissue remodeling. Rheumatoid arthritis (RA) exemplifies this advanced stage. Its hallmark features, including persistent synovitis, pannus formation, and progressive bone destruction, parallel malignant tumors in local invasion and dysregulated growth. Overlap analysis identified 123 shared targets between Osthole and RA (Figure 5A). Compared with the preceding barrier-centered models, the RA PPI network appeared more extensive and interconnected, with inflammatory, apoptotic, and remodeling-associated nodes, including RELA, AKT1, CASP3, PTGS2, and MMP9, occupying highly connected regions (Figure 5B). This distribution suggests that the Osthole-associated target set in RA may be linked to inflammatory amplification, kinase signaling, cell-fate regulation, and matrix-destructive remodeling.

Functional enrichment analysis (GO and KEGG; Figure 5C,D) indicated enrichment in the regulation of apoptotic signaling, responses to bacterial molecules, serine/threonine kinase activities, and transcription factor binding. Enrichment of extrinsic apoptotic signaling, cytokine receptor binding, and MAPK/JUN kinase activity was compatible with the inflammatory and invasive characteristics of the synovial microenvironment. Collectively, these findings place the RA-related targets within a network shaped by persistent inflammation, apoptotic imbalance, and destructive tissue remodeling. PTGS2 (COX-2) was prioritized for docking because of its role in inflammatory mediator production and local tissue injury. Docking positioned Osthole within the PTGS2 binding region, with a calculated binding energy of −8.6 kcal/mol, and key predicted contacts included ALA-202, THR-206, and TYR-385 (Table 2 and Figure 5E).

#### 3.4.4. Damage-Driven Inflammation at the Threshold of Maladaptive Repair

**Figure 6:**
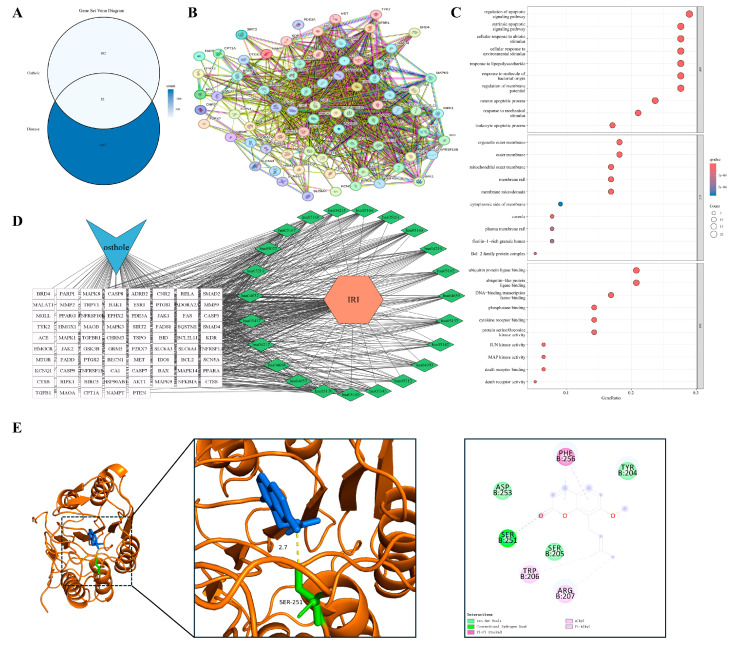
Network pharmacology and molecular docking analysis of Osthole in IRI: (**A**) Venn diagram showing the intersection between predicted Osthole targets and IRI-associated genes; 83 overlapping targets were retained for further analysis. (**B**) PPI network of the overlapping targets generated using STRING and visualized in Cytoscape. (**C**) GO enrichment analysis of the overlapping targets, including BP, CC, and MF categories. Dot size represents gene count, and dot color represents enrichment significance. (**D**) Drug–target–disease–pathway network showing predicted associations among Osthole, overlapping targets, enriched KEGG pathways, and IRI. (**E**) Docking analysis of Osthole with CASP3 (PDB ID: 1GFW), including the overall 3D binding pose, magnified binding pocket, and 2D ligand–residue interaction diagram.

Beyond chronic inflammatory invasion, acute inflammatory cascades triggered by tissue injury also drive pathological evolution. Renal ischemia–reperfusion injury (IRI) is a prototypical model of injury-induced sterile inflammation, in which parenchymal cell necrosis releases damage-associated molecular patterns (DAMPs), such as HMGB1, contributing to secondary inflammation and maladaptive repair responses. Overlap analysis identified 83 shared targets between Osthole and IRI (Figure 6A). In contrast to the preceding chronic remodeling models, the IRI PPI network showed coexistence of cell-death regulators and inflammatory mediators. Representative nodes, including CASP3, BAX, BCL2, RELA, RIPK1, and HMGB1, were embedded in the interconnected network (Figure 6B). This pattern is consistent with the injury-driven nature of IRI, where apoptosis and inflammatory amplification are closely coupled.

Enrichment analysis (Figure 6C,D) indicated that these targets were enriched in pathways governing apoptotic signaling, responses to abiotic stimuli, MAPK-related kinase activity, and death receptor binding. Terms related to extrinsic apoptotic signaling, regulation of membrane potential, and death receptor functions were particularly relevant to acute tubular injury and secondary inflammatory activation. Thus, the IRI model may highlight a damage-response module linking cell death and sterile inflammation. CASP3 was selected as the representative docking target because of its role in the execution phase of apoptosis. Docking placed Osthole within the CASP3 binding region, with a calculated binding energy of −6.2 kcal/mol, and the predicted interaction involved SER-251 as a key residue (Table 2 and Figure 6E).

#### 3.4.5. Persistent Neuroinflammation in a Distinct Tissue Context

**Figure 7:**
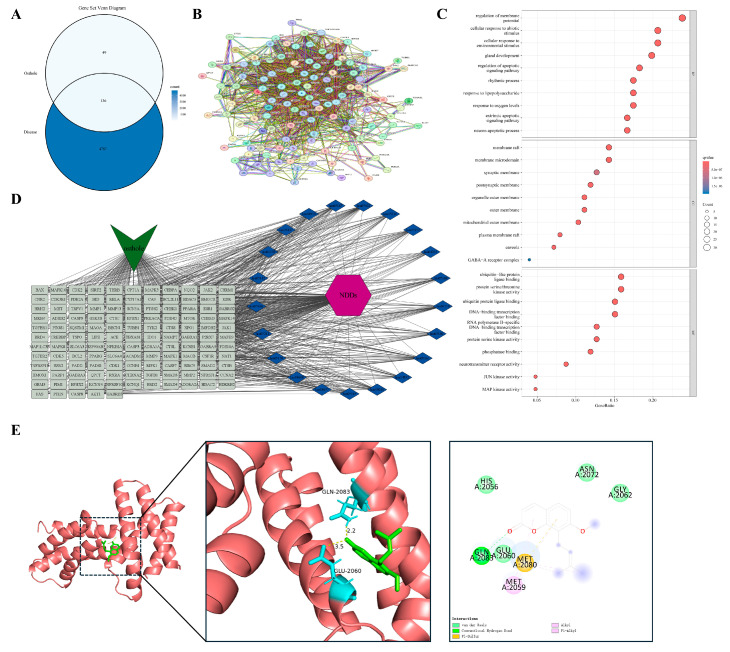
Network pharmacology and molecular docking analysis of Osthole in neurodegenerative diseases: (**A**) Venn diagram showing the intersection between predicted Osthole targets and neurodegenerative disease-associated genes; 136 overlapping targets were retained for further analysis. (**B**) PPI network of the overlapping targets generated using STRING and visualized in Cytoscape. (**C**) GO enrichment analysis of the overlapping targets, including BP, CC, and MF categories. Dot size represents gene count, and dot color represents enrichment significance. (**D**) Drug–target–disease–pathway network showing predicted associations among Osthole, overlapping targets, enriched KEGG pathways, and neurodegenerative diseases. (**E**) Docking analysis of Osthole with MTOR (PDB ID: 1AUE), including the overall 3D binding pose, magnified binding pocket, and 2D ligand–residue interaction diagram.

Chronic neuroinflammation within the central nervous system constitutes a distinct pathological milieu that differs from peripheral tissue inflammation and remodeling. Neurodegenerative diseases are characterized by persistent microglial activation and sustained release of neurotoxic mediators. This unresolved inflammatory state, which lacks an effective termination mechanism, contributes to progressive neuronal loss and the accumulation of pathogenic protein aggregates. We identified 136 shared targets between Osthole and neurodegenerative disease-related genes (Figure 7A). The PPI network was highly connected, but unlike the peripheral models, several representative nodes were associated with neuronal stress adaptation and metabolic regulation, including MTOR, AKT1, MAPK3, RELA, and CASP3 (Figure 7B). This distribution suggests that the Osthole-associated target set in neurodegeneration may be linked not only to inflammatory signaling but also to neuronal survival and stress-response pathways.

Enrichment analysis (Figure 7C,D) indicated enrichment in stress responses to hypoxia, lipopolysaccharide, and other stimuli, together with regulation of apoptotic signaling and serine/threonine kinase-dependent pathways, including MAPK and JNK. Subcellular localization analysis further indicated enrichment in the mitochondrial outer membrane, postsynaptic membrane, lipid rafts, GABA-A receptor complex, and neurotransmitter receptor activity. These terms distinguished this model from peripheral tissue models and linked the neurodegenerative disease-related target set to neuroinflammatory stress, synaptic regulation, and neuronal survival. MTOR was chosen as the representative docking target because of its role in autophagy, energy metabolism, and cell growth. Docking predicted an Osthole–MTOR interaction with a calculated binding energy of −7.9 kcal/mol and key contacts involving LYS-2046 and VAL-2045 (Table 2 and Figure 7E).

#### 3.4.6. When Persistent Inflammation Becomes Fibrosis

**Figure 8:**
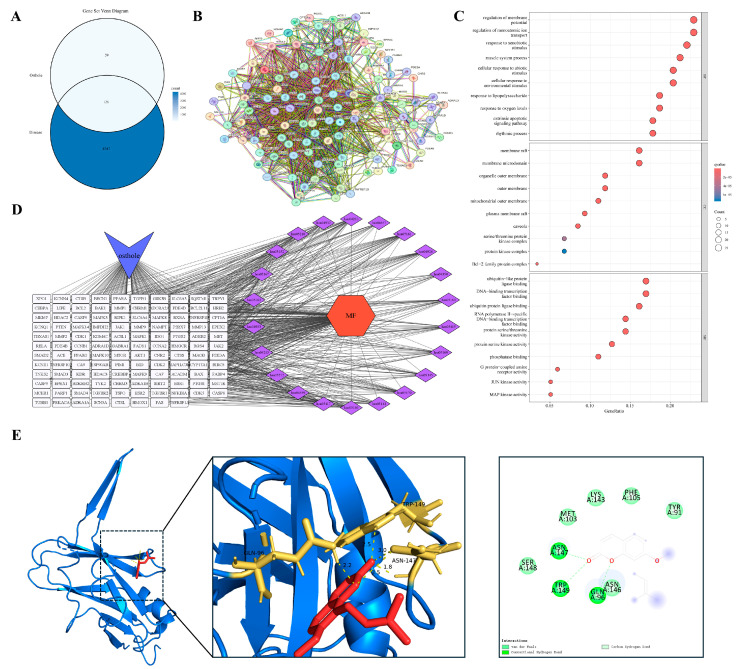
Network pharmacology and molecular docking analysis of Osthole in myocardial fibrosis: (**A**) Venn diagram showing the intersection between predicted Osthole targets and myocardial fibrosis-associated genes; 126 overlapping targets were retained for further analysis. (**B**) PPI network of the overlapping targets generated using STRING and visualized in Cytoscape. (**C**) GO enrichment analysis of the overlapping targets, including BP, CC, and MF categories. Dot size represents gene count, and dot color represents enrichment significance. (**D**) Drug–target–disease–pathway network showing predicted associations among Osthole, overlapping targets, enriched KEGG pathways, and myocardial fibrosis. (**E**) Docking analysis of Osthole with TGFB1 (PDB ID: 1KLC), including the overall 3D binding pose, magnified binding pocket, and 2D ligand–residue interaction diagram.

Myocardial fibrosis, a critical sequela of chronic inflammation or prolonged hemodynamic pressure overload, exemplifies maladaptive tissue repair. This condition features sustained fibroblast activation and deleterious accumulation of extracellular matrix components. Its core signaling drivers, particularly the TGF-β pathways, show mechanistic overlap with the matrix remodeling observed in the tumor microenvironment. Overlap analysis identified 126 shared targets between Osthole and myocardial fibrosis (Figure 8A). The PPI network was compact, with several fibrosis- and stress-associated nodes, including TGFB1, SMAD2, AKT1, MAPK1/3, and BCL2, embedded within the central network region (Figure 8B). Compared with the acute injury model, this distribution emphasized profibrotic signaling and sustained remodeling rather than immediate damage-response signals.

Enrichment analysis (Figure 8C and Figure 8D) indicated that these targets were primarily concentrated in membrane potential regulation, responses to exogenous and abiotic stimuli, and MAPK- or JNK-dependent serine/threonine kinase activities. Enrichment of ion transport, muscle system process, and kinase complex terms reflected the contractile and electrophysiological background of myocardial remodeling. Subcellular localization indicated enrichment within the mitochondrial outer membrane, lipid rafts, and kinase complexes. Together, these results connect the myocardial fibrosis-related target set with ion handling, kinase signaling, and sustained matrix remodeling. TGFB1 was selected for docking because of its established role in fibrogenesis and tissue remodeling. Docking predicted a favorable Osthole-binding pose within TGFB1, with a calculated binding energy of −7.7 kcal/mol and key contacts involving GLN-96, ASN-147, and TRP-149 (Table 2 and Figure 8E).

#### 3.4.7. A Convergent Trajectory from Inflammation to Remodeling

Pathological models across diverse organ systems indicate that the transition from persistent inflammatory stress to maladaptive tissue remodeling represents a highly conserved disease trajectory. From ischemia-induced parenchymal necrosis and chronic neuroinflammation to profibrotic myocardial remodeling, core regulatory networks consistently converge on the PI3K/Akt, NF-κB, and TGF-β/Smad signaling pathways. By potentially engaging these cascades, Osthole may have therapeutic relevance beyond disease-specific symptomatic relief. Instead, it may be associated with the modulation of inflammatory amplification, tissue homeostasis, and aberrant repair processes. Ultimately, these predicted regulatory mechanisms provide a computational framework for understanding the potential pharmacological basis of Osthole in inflammation-driven tissue remodeling and malignant transformation.

### 3.5. From Tissue Remodeling to Inflammation-Associated Malignancy

#### 3.5.1. Inflammation-Driven Malignant Transformation and the Hepatocellular Carcinoma Microenvironment

**Figure 9:**
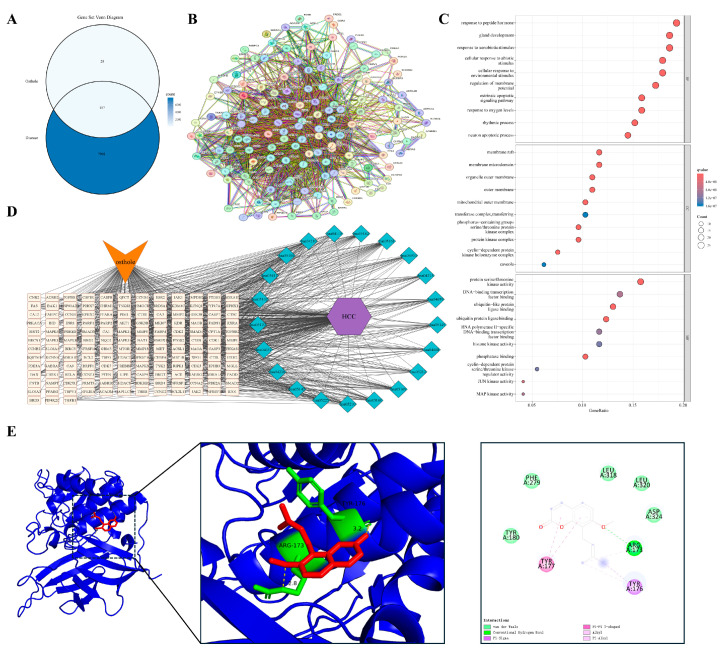
Network pharmacology and molecular docking analysis of Osthole in HCC: (**A**) Venn diagram showing the intersection between predicted Osthole targets and HCC-associated genes; 157 overlapping targets were retained for further analysis. (**B**) PPI network of the overlapping targets generated using STRING and visualized in Cytoscape. (**C**) GO enrichment analysis of the overlapping targets, including BP, CC, and MF categories. Dot size represents gene count, and dot color represents enrichment significance. (**D**) Drug–target–disease–pathway network showing predicted associations among Osthole, overlapping targets, enriched KEGG pathways, and HCC. (**E**) Docking analysis of Osthole with PTEN (PDB ID: 1D5R), including the overall 3D binding pose, magnified binding pocket, and 2D ligand–residue interaction diagram.

Hepatocellular carcinoma (HCC) is a paradigmatic model within the inflammation–cancer axis, typically arising from persistent hepatic inflammation and tissue injury due to viral infection, chronic alcohol consumption, or metabolic stress. Intersection analysis identified 157 shared targets between Osthole and HCC (Figure 9A). The PPI network was highly interconnected, with representative nodes such as PTEN, AKT1, RELA, and CASP3 positioned within the central interaction landscape, suggesting that the Osthole-associated target set in HCC may be associated with survival signaling, inflammatory activity, and cell-fate control (Figure 9B).

Enrichment analysis (Figure 9C,D) indicated enrichment in pathways governing responses to environmental and exogenous stimuli, regulation of extrinsic apoptotic signaling, and processes involving histone kinases and ubiquitin ligase binding. Subcellular localization analysis indicated association with the mitochondrial outer membrane, lipid rafts, and serine/threonine kinase complexes. The enrichment of ubiquitin ligase binding and histone kinase-related functions distinguished this model from non-neoplastic disease settings, consistent with the regulatory complexity of malignant transformation. These findings connect the HCC-related target set with proliferative signaling, stress adaptation, and programmed cell death. PTEN was chosen as the representative docking target because of its critical tumor-suppressor function in antagonizing aberrant PI3K/Akt activation. Molecular docking suggested a plausible Osthole-binding pose for PTEN, with a calculated binding energy of −7.3 kcal/mol and key contacts involving ARG-173 and TYR-176 (Table 2 and Figure 9E).

#### 3.5.2. Malignancy Arising on a Background of Chronic Airway Remodeling

**Figure 10:**
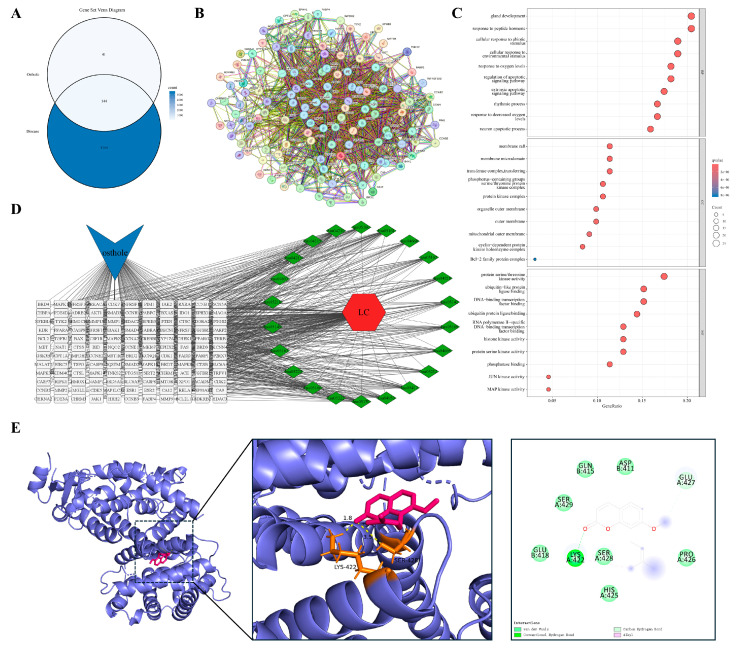
Network pharmacology and molecular docking analysis of Osthole in lung cancer: (**A**) Venn diagram showing the intersection between predicted Osthole targets and lung cancer-associated genes; 144 overlapping targets were retained for further analysis. (**B**) PPI network of the overlapping targets generated using STRING and visualized in Cytoscape. (**C**) GO enrichment analysis of the overlapping targets, including BP, CC, and MF categories. Dot size represents gene count, and dot color represents enrichment significance. (**D**) Drug–target–disease–pathway network showing predicted associations among Osthole, overlapping targets, enriched KEGG pathways, and lung cancer. (**E**) Docking analysis of Osthole with PPARG (PDB ID: 2Q59), including the overall 3D binding pose, magnified binding pocket, and 2D ligand–residue interaction diagram.

Persistent airway inflammation resulting from long-term smoking or chronic obstructive pulmonary disease is a major driver of pulmonary oncogenesis. In the lung cancer model, we identified 144 shared targets between Osthole and lung cancer (Figure 10A). The PPI network was closely connected, with PPARG, AKT1, RELA, and CASP3 positioned in the main interaction framework (Figure 10B), linking the Osthole-associated target set to inflammatory signaling, stress adaptation, and cell survival control.

Enrichment analysis (Figure 10C,D) indicated enrichment in cellular responses to abiotic stressors, including fluctuations in oxygen tension, regulation of extrinsic apoptotic signaling, and the activity of serine/threonine kinases and DNA-binding transcription factors. Subcellular localization analysis indicated enrichment in structures essential for cell survival and death, most notably Bcl-2 family protein complexes and lipid rafts. Terms related to oxygen tension and Bcl-2 family complexes were consistent with the hypoxia-adaptive and apoptosis-resistant characteristics of the lung cancer microenvironment. Together, these findings place the lung cancer-related target set within pathways associated with hypoxic stress, inflammatory signaling, and apoptosis resistance. PPARG was selected as the representative docking target because of its role in lipid metabolism, resolution of inflammation, and cellular differentiation. Docking suggested a compatible Osthole-binding mode, with a calculated binding energy of −8.0 kcal/mol and key contacts involving LYS-422 and SER-428 (Table 2 and Figure 10E).

#### 3.5.3. Repeated Injury and Repair in an Inflammation-Prone Malignancy

**Figure 11:**
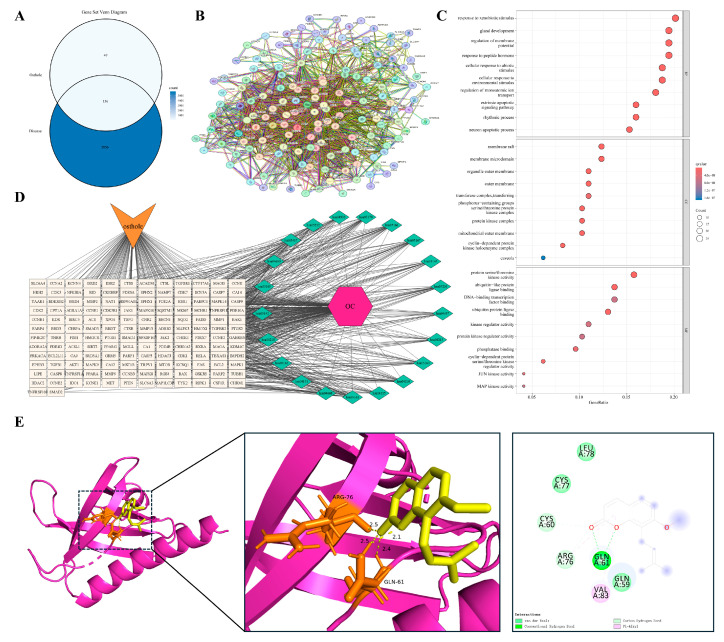
Network pharmacology and molecular docking analysis of Osthole in ovarian cancer: (**A**) Venn diagram showing the intersection between predicted Osthole targets and ovarian cancer-associated genes; 136 overlapping targets were retained for further analysis. (**B**) PPI network of the overlapping targets generated using STRING and visualized in Cytoscape. (**C**) GO enrichment analysis of the overlapping targets, including BP, CC, and MF categories. Dot size represents gene count, and dot color represents enrichment significance. (**D**) Drug–target–disease–pathway network showing predicted associations among Osthole, overlapping targets, enriched KEGG pathways, and ovarian cancer. (**E**) Docking analysis of Osthole with AKT1 (PDB ID: 3O96), including the overall 3D binding pose, magnified binding pocket, and 2D ligand–residue interaction diagram.

Ovarian cancer is a unique pathological model in which malignant transformation is closely linked to recurrent cycles of ovulation-induced tissue injury and repair. Persistent local inflammatory cell infiltration and proinflammatory mediators are thought to foster a microenvironment conducive to genomic instability and cellular transformation. Overlap analysis identified 136 shared targets between Osthole and ovarian cancer (Figure 11A). The PPI network was dense, with representative nodes such as AKT1, CASP3, RELA, and CDK1 incorporated into the main network architecture (Figure 11B), consistent with the involvement of survival signaling, inflammatory regulation, apoptotic execution, and cell-cycle control.

Enrichment analysis (Figure 11C,D) indicated enrichment in pathways governing responses to exogenous stimuli, regulation of membrane potential and ion transport, and activities of serine/threonine kinases and transcription factors. Subcellular localization analysis indicated enrichment in lipid rafts, mitochondrial outer membranes, and cyclin-dependent kinase complexes, suggesting that the Osthole-associated target network spans environmental stress adaptation, cell-cycle regulation, and survival control. Among these features, membrane potential regulation, ion transport, and CDK complex localization were especially relevant to the recurrent injury-repair background and proliferative phenotype of ovarian cancer. This profile links the ovarian cancer-related target set to ion homeostasis, cell-cycle progression, and survival signaling within a repeatedly injured inflammatory niche. AKT1 was selected for docking given its central role in cell survival, anti-apoptotic programs, and metabolic reprogramming. Docking predicted an Osthole-binding mode with a calculated binding energy of −8.3 kcal/mol and key contacts involving ARG-76 and GLN-61 (Table 2 and Figure 11E).

#### 3.5.4. Malignant Endpoints of a Shared Pathological Continuum

Evidence from diverse disease models, including inflammatory conditions and solid tumors investigated in cell lines, animal models, and xenografts under varying doses and endpoints, nonetheless points to the recurrent involvement of PI3K/Akt and NF-κB signaling in Osthole-related pharmacological responses. As summarized in Table 3, Osthole has repeatedly been reported to arrest cell-cycle progression, induce mitochondria-mediated apoptosis, and attenuate invasive matrix remodeling across both non-malignant and malignant contexts. This mechanistic convergence, despite the inherent heterogeneity of the primary data, suggests an intersection between the inflammatory cascades that drive early-stage tissue remodeling and the molecular programs that sustain late-stage neoplastic progression. Accordingly, these findings support the potential relevance of Osthole as an intervention candidate within the inflammation-remodeling-malignancy spectrum and provide a rationale for further investigation of its systemic effects on the inflammation–cancer axis.

### 3.6. Osthole Maintains Robust Atomic-Level Stability Across Key Signaling Hubs

To validate the conformational stability of Osthole binding and characterize its interaction dynamics in a solvated environment, we prioritized high-confidence core targets from the integrated PPI network. The selection was based on a rigorous synthesis of signaling pathway representation, docking affinity, and their respective roles within the inflammation–cancer axis. Consequently, AKT1, RELA, and TGFB1 were identified as the most representative targets for MD simulations. These three nodes encapsulate the critical functional pillars of the disease process: the PI3K/Akt-mediated survival pathway, the NF-κB-driven inflammatory axis, and the TGF-β/Smad-mediated module governing fibrosis and metastasis. Accordingly, we interpreted the MD trajectories not only as indicators of binding stability, but also in relation to the known functional requirements of each target: kinase activation for AKT1, transcriptional regulation for RELA, and receptor engagement for TGFB1. To provide a more quantitative comparison of the three simulated systems, the key post-equilibration MD parameters, including complex RMSD, ligand RMSD, RMSF, Rg, and SASA, are summarized in Table 4.

#### 3.6.1. Rapid Convergence and Structural Integrity of the Osthole-AKT1 Complex

**Figure 12:**
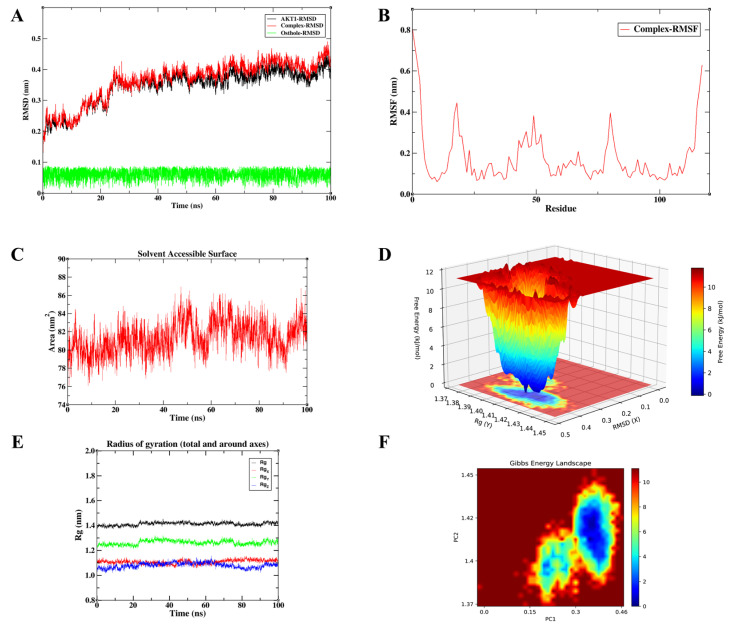
MD simulation analysis of the Osthole–AKT1 complex using GROMACS 2025.2 for a 100 ns simulation: (**A**) RMSD trajectories illustrating the dynamic stability of the protein backbone, complex, and ligand. (**B**) RMSF profile indicating residue-level flexibility of AKT1. (**C**) SASA profile reflecting changes in solvent exposure during MD simulation. (**D**) 3D Gibbs FEL projected onto RMSD and Rg. (**E**) Total Rg and axial Rg distributions representing the compactness of the complex. (**F**) 2D FEL based on PC1 and PC2 from PCA.

Figure 12 illustrates the MD simulation profiles for the Osthole-AKT1 complex. The RMSD trajectories showed an initial conformational adjustment during the first approximately 25 ns, after which the protein and complex RMSD values entered a relatively stable plateau. During the equilibrated phase from 25 to 100 ns, the AKT1 protein RMSD was 0.374 ± 0.022 nm, while the complex RMSD was 0.396 ± 0.029 nm, with values mainly fluctuating within 0.35–0.45 nm. Notably, the Osthole RMSD remained low throughout the equilibrated trajectory, with a mean value of 0.060 ± 0.017 nm and a maximum value below 0.10 nm, suggesting limited positional displacement of the ligand within the predicted AKT1 binding region. RMSF analysis showed an average fluctuation of 0.175 ± 0.128 nm across all residues. The higher RMSF values were mainly located at the terminal regions and several flexible loop segments, whereas the non-terminal region showed a lower average RMSF of 0.149 ± 0.079 nm. Furthermore, the total Rg remained stable at 1.417 ± 0.011 nm during 25–100 ns, supporting the maintenance of global structural compactness. The SASA value was 81.780 ± 1.663 nm^2^ over the same interval, indicating limited variation in solvent exposure rather than major unfolding or structural expansion. Combined with the single, well-defined low-energy basin observed in the Gibbs free energy landscape, these findings indicate that the complex remains in a compact and stable conformational state. This is relevant to AKT1 function because kinase activation depends on coordinated local motions that support phosphorylation, ATP binding, and substrate recognition. Sustained retention of Osthole within the predicted AKT1 binding region may reduce this conformational freedom and, in turn, weaken signal transfer through the PI3K/Akt axis. The stable AKT1-bound state observed here therefore provides structural support for the hypothesis that Osthole may interact with AKT1 within the PI3K/Akt-related signaling axis.

#### 3.6.2. Osthole Binding Restricts the Conformational Flexibility of RELA

**Figure 13:**
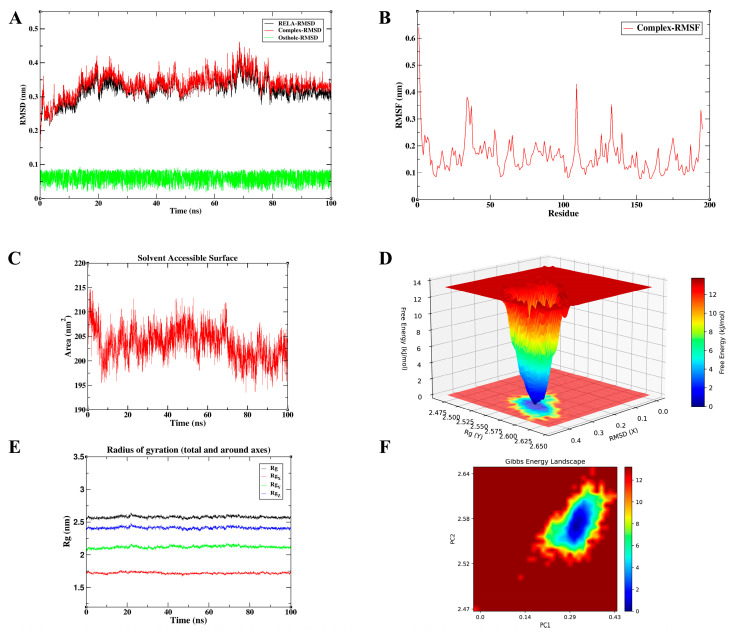
MD simulation analysis of the Osthole–RELA complex using GROMACS 2025.2 for a 100 ns simulation: (**A**) RMSD trajectories illustrating the dynamic stability of the protein backbone, complex, and ligand. (**B**) RMSF profile indicating residue-level flexibility of RELA. (**C**) SASA profile reflecting changes in solvent exposure during MD simulation. (**D**) 3D Gibbs FEL projected onto RMSD and Rg. (**E**) Total Rg and axial Rg distributions representing the compactness of the complex. (**F**) 2D FEL based on PC1 and PC2 from PCA.

The 100 ns MD trajectory for the Osthole-RELA complex demonstrates conformational stability (Figure 13). Following an initial equilibration phase of approximately 20 ns, both the RELA protein and complex RMSD values entered a relatively stable plateau. During the equilibrated phase from 20 to 100 ns, the protein RMSD was 0.332 ± 0.026 nm, while the complex RMSD was 0.346 ± 0.025 nm, with values primarily fluctuating within 0.30–0.45 nm. The Osthole RMSD remained low throughout the equilibrated trajectory (0.060 ± 0.017 nm; max 0.091 nm), suggesting limited positional displacement within the predicted RELA binding pocket. RMSF analysis showed an average fluctuation of 0.162 ± 0.068 nm across all residues, with higher fluctuations mainly at the N-terminal and solvent-exposed loop regions. Excluding terminal residues, the average RMSF was 0.157 ± 0.056 nm, indicating that the main structural core remained relatively stable. The radius of gyration remained approximately 2.577 ± 0.016 nm, and SASA values were 203.452 ± 3.120 nm^2^, supporting the maintenance of global structural compactness without substantial expansion. Notably, the well-defined energy well in the FEL analysis suggests that Osthole stabilizes RELA in a low-energy conformational state. RELA/p65 requires structural flexibility to interact with regulatory proteins, assemble transcriptional complexes, and sustain NF-κB-dependent gene expression. Reduced flexibility around the predicted binding region may therefore limit the conformational adaptability needed for full RELA activity. Taken together, the stable low-energy RELA-bound state observed here supports the structural plausibility of the Osthole–RELA interaction and its potential relevance to NF-κB-related inflammatory signaling.

#### 3.6.3. Exceptional Rigidity and Compactness of the Osthole–TGFB1 Interaction

**Figure 14:**
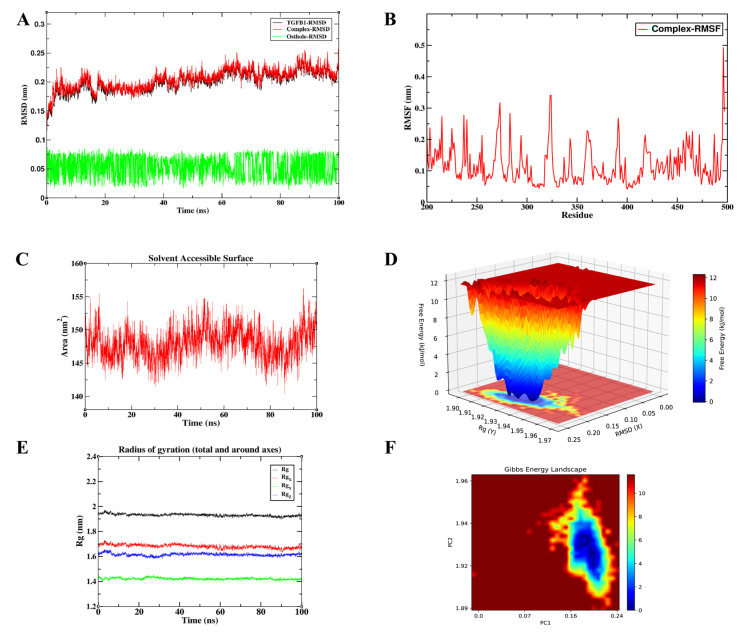
MD simulation analysis of the Osthole–TGFB1 complex using GROMACS 2025.2 for a 100 ns simulation: (**A**) RMSD trajectories illustrating the dynamic stability of the protein backbone, complex, and ligand. (**B**) RMSF profile indicating residue-level flexibility of TGFB1. (**C**) SASA profile reflecting changes in solvent exposure during MD simulation. (**D**) 3D Gibbs FEL projected onto RMSD and Rg. (**E**) Total Rg and axial Rg distributions representing the compactness of the complex. (**F**) 2D FEL based on PC1 and PC2 from PCA.

Representing the central node of the fibrosis and metastasis module, the Osthole-TGFB1 complex exhibited the most stable dynamic profile among the tested systems (Figure 14). As shown in Table 4, this complex displayed the lowest protein RMSD, complex RMSD, ligand RMSD, and RMSF values among the three simulated systems. The system reached an apparent conformational equilibrium within approximately 10 ns. During the equilibrated phase from 10 to 100 ns, the TGFB1 protein RMSD was 0.204 ± 0.015 nm, while the complex RMSD was 0.208 ± 0.015 nm, with values mainly fluctuating within 0.17–0.26 nm. The Osthole RMSD remained low throughout the equilibrated trajectory, with a mean value of 0.054 ± 0.018 nm and a maximum value of 0.086 nm, suggesting limited ligand displacement within the predicted TGFB1 binding region. RMSF analysis showed an average fluctuation of 0.117 ± 0.062 nm across all residues, and the average RMSF remained 0.115 ± 0.057 nm after excluding terminal residues, indicating restricted local flexibility in the main structural region. Furthermore, the radius of gyration remained stable at 1.930 ± 0.009 nm, and the SASA value was 147.995 ± 2.287 nm^2^ during 10–100 ns, indicating that the complex maintained a compact global conformation without marked structural expansion or solvent-exposure variation. Together with the single-basin energy profile in the FEL map, these quantitative parameters support a stable Osthole-TGFB1 association. Among the three simulated systems, this complex showed the lowest RMSD and RMSF values, suggesting the greatest conformational stability under the present simulation conditions. This pattern raises the possibility that Osthole may reduce the conformational availability of TGFB1 for productive receptor engagement. In a cellular setting, such an effect could weaken downstream Smad2/3 activation and reduce TGF-β-driven fibrotic or EMT-related responses. Taken together, the relatively stable and low-energy TGFB1-bound state observed here supports the structural plausibility of the Osthole–TGFB1 interaction and its potential relevance to TGF-β/Smad-related fibrotic and EMT-associated signaling.

#### 3.6.4. Hydrophobic Forces Drive the High-Affinity Multi-Target Binding of Osthole

**Figure 15:**
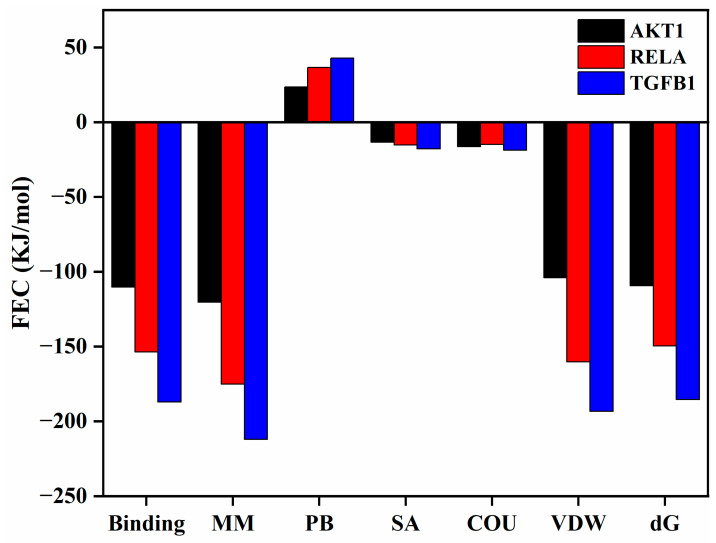
MM/PBSA-based binding energy decomposition of the Osthole-target complexes: Binding energy estimates for Osthole-AKT1, Osthole-RELA, and Osthole-TGFB1 were calculated using gmx_MMPBSA v1.6.1 and decomposed into molecular mechanics energy, vdW contribution, electrostatic contribution, polar solvation energy, and non-polar solvation energy. Negative and positive values indicate favorable and unfavorable energetic contributions, respectively.

To quantitatively evaluate the binding affinities from a thermodynamic perspective, MM/PBSA calculations were performed on the equilibrated 100 ns trajectories (Figure 15). The total binding free energies (ΔG) were highly favorable for all three hub targets: −109.273 KJ/mol for AKT1, −149.536 KJ/mol for RELA, and −185.472 KJ/mol for TGFB1. These values support the possibility that Osthole may form energetically favorable associations with the key signaling nodes. Per-residue energy decomposition further revealed that van der Waals (VDW) interactions and non-polar solvation (SA) contributions provided the dominant favorable forces (VDW: −104.028, −160.238, and −193.406 KJ/mol; SA: −13.335, −15.234, and −17.743 KJ/mol for AKT1, RELA, and TGFB1, respectively), indicating that the hydrophobic coumarin ring of Osthole is ideally sequestered within the target binding pockets. In contrast, the polar solvation (PB) term imposed a predictable energy penalty (23.499, 36.673, and 42.799 KJ/mol), while the gas-phase molecular mechanics energy (MM) was strongly negative. The minimum binding energy observed for the TGFB1 complex also corresponded directly with its superior conformational stability in the MD analysis. These thermodynamic profiles strengthen the structural rationale for Osthole’s predicted multi-target interactions, and provide a basis for further investigating how these predicted molecular interactions may relate to systemic modulation of the inflammation–cancer axis.

## 4. Discussion

Natural products are increasingly recognized as a useful source of multi-target agents for complex diseases involving interconnected signaling networks. This perspective is particularly relevant to chronic inflammation and cancer, which share multiple regulatory pathways and biological processes [66,67,68,69]. Osthole, a coumarin compound derived from Cnidium monnieri, has been reported to exhibit both anti-inflammatory and antitumor activities; however, the relationship between these effects has remained insufficiently defined. In the present work, we combined network pharmacology, molecular docking, molecular dynamics simulation, and literature-based evidence synthesis to examine whether the currently available data supports a shared mechanistic framework. The results indicate that the pharmacological actions of Osthole may converge on several central signaling pathways, particularly PI3K/Akt, NF-κB, and TGF-β/Smad, all of which are implicated in both inflammatory and oncogenic processes. More importantly, our dynamic microscopic-level structural analyses provided support for the plausibility of this mechanistic convergence.

Among these pathways, PI3K/Akt appears to be one of the most consistent nodes linking the anti-inflammatory and antitumor activities of Osthole. This pathway plays an important role in immune-cell activation, survival signaling, and metabolic regulation [70]. In our analysis, PI3K/Akt signaling was significantly enriched among the core targets of Osthole, and molecular docking suggested a potential interaction between Osthole and AKT1. The molecular dynamics results further strengthened this observation: the Osthole–AKT1 complex showed stable conformational behavior, favorable binding affinity, and a single dominant low-energy basin, which supports the possibility that Osthole may directly engage AKT1 and modulate PI3K/Akt signaling. This interpretation is consistent with previous experimental studies showing that Osthole suppresses PI3K activation and catalytic activity, thereby reducing AKT-related signaling [71]. In inflammatory settings, phosphorylated AKT can downregulate ZO-3, a tight-junction protein involved in epithelial barrier repair, whereas Osthole has been shown to inhibit AKT phosphorylation, restore ZO-3 expression, and promote the repair of inflammatory skin injury [38,72]. This regulatory axis is exemplified in the skin, where Osthole-mediated inhibition of AKT phosphorylation restores ZO-3 expression and promotes cutaneous barrier repair [38,72,73], directly linking PI3K/Akt signaling to tissue integrity restoration. In cancer models, aberrant AKT activation supports tumor-cell proliferation and survival, and its inhibition by Osthole has been associated with reduced tumor growth and increased apoptosis [74]. AKT also acts upstream of NF-κB by promoting IκB degradation and enhancing IKKα and Tpl2 phosphorylation; therefore, suppression of AKT activity may also limit AKT/NF-κB signaling, reducing both inflammatory responses and NF-κB-driven protumor programs [75,76]. In addition, P-gp, a major efflux transporter involved in multidrug resistance, is functionally linked to PI3K/Akt signaling. The reported ability of Osthole to inhibit both AKT activity and P-gp function suggests that this axis may also contribute to chemosensitization in selected tumor contexts [65,77]. This mechanistic continuity is particularly evident along the respiratory disease continuum: Osthole targets the same PI3K/Akt and NF-κB axes in both chronic airway inflammation and lung cancer, suggesting that its anti-inflammatory and anticancer activities are functionally linked through shared signaling nodes. Thus, the computational results and published experimental evidence together support a role for PI3K/Akt signaling in the anti-inflammatory, anticancer, and potential chemosensitizing effects of Osthole. However, PI3K/Akt should not be viewed as a single dominant mechanism. Rather, it is best interpreted as one representative shared signaling axis through which Osthole may act across different pathological settings [78,79]. Further studies are needed to delineate the specific pathological contexts in which this axis predominates.

NF-κB signaling also showed a strong integrative role in bridging chronic inflammation and tumor promotion. NF-κB is widely recognized as a central link between chronic inflammation and tumor-promoting processes. It regulates the initiation and maintenance of immune and inflammatory responses, while also supporting cancer-related processes such as cell proliferation, resistance to apoptosis, angiogenesis, and metastasis [80]. In our KEGG analysis, the NF-κB signaling pathway was significantly enriched, and RELA, which encodes the p65 transcriptional subunit of NF-κB, was identified as a core target. At the molecular level, Osthole formed a compact and stable complex with RELA in our molecular dynamics simulations, supporting the possibility that Osthole may interfere with abnormal NF-κB activation. Experimental evidence is consistent with this interpretation, showing that Osthole may reduce NF-κB activation by delaying IκBα degradation, limiting NF-κB nuclear translocation, and suppressing downstream transcriptional activity [81,82,83]. This mechanism is biologically relevant because IκBα masks the nuclear localization signal of NF-κB and retains it in the cytoplasm, whereas NF-κB nuclear translocation is required for the transcriptional activation of many pro-inflammatory and pro-tumor genes [41,84]. In addition, Osthole has been reported to activate the Nrf2 antioxidant pathway, which functionally intersects with NF-κB signaling [85,86]. Nrf2 can regulate gene expression through antioxidant response elements and reduce the production of NF-κB-driven inflammatory mediators, including IL-6 and TNF-α [87,88]. Kobayashi et al. further showed that Nrf2 can suppress the transcriptional upregulation of pro-inflammatory NF-κB target genes independently of its redox-regulatory function [89]. Importantly, this Nrf2/NF-κB regulatory network is not restricted to peripheral tissues. In the CNS, Osthole has been shown to activate Nrf2 signaling in microglia, thereby suppressing NF-κB-driven neuroinflammation [51,90], suggesting that the same mechanistic framework operates across diverse tissue environments. This intersection is particularly important because IL-6 and TNF-α are also important tumor-promoting cytokines in the tumor microenvironment; the Nrf2–NF-κB crosstalk induced by Osthole may help connect its anti-inflammatory activity with its effects on inflammation-associated tumor progression [91]. Taken together, these lines of evidence converge to support the involvement of NF-κB signaling in the anti-inflammatory and anticancer activities of Osthole. However, the current evidence is not sufficient to conclude that NF-κB alone represents a unified mechanism across all disease models discussed here. It should instead be viewed as one shared signaling node that requires further validation in specific pathological contexts.

TGF-β/Smad signaling provides another explanation for the mechanistic overlap between chronic inflammatory remodeling and tumor progression. Sustained activation of this pathway can promote organ fibrosis, epithelial–mesenchymal transition (EMT), and metastatic behavior, although TGF-β signaling is also essential for tissue repair and immune homeostasis [92,93,94]. In our analysis, TGFB1 was identified as a core target, and enrichment analysis pointed to the involvement of TGF-β-related pathways. Molecular docking further predicted a potential direct interaction between Osthole and TGFB1, suggesting that Osthole may affect this pathway at the ligand level. In fibrotic conditions such as myocardial fibrosis and asthmatic airway remodeling, TGF-β1 activates Smad2/3 and stimulates fibroblast activation and extracellular matrix deposition, thereby contributing to tissue remodeling [95]. TGFB1 is also a key mediator in immune-mediated inflammatory disease, and activation of Smad2/3 can promote interstitial fibrosis and organ injury [96]. Our molecular dynamics simulations showed that the Osthole–TGFB1 complex had marked structural rigidity and binding cohesion, providing a molecular basis for the reported inhibition of Smad2/3 phosphorylation and downstream profibrotic signaling [50,97]. Consistent with this view, experimental studies have shown that Osthole can suppress Smad2/3 activation and reduce downstream mediators such as p38, NF-κB, and IL-1 [98,99,100]. This mechanism may also contribute to inhibition of epithelial–mesenchymal transition and cancer cell dissemination, given the established role of TGF-β as a potent inducer of this process [101,102]. This aligns with earlier evidence that the TGF-β/Smad pathway is a central driver of EMT and that EMT is a key step in the acquisition of tumor-cell invasion and metastatic potential [103,104,105]. Osthole-mediated blockade of Smad signaling has also been linked to reduced cancer-cell migration and invasion [106]. Furthermore, Smad inhibition may further limit the activation of downstream mediators, including p38 MAPK and NF-κB, suggesting broader relevance in tumors with dysregulated TGF-β signaling [107]. Taken together, these findings indicate that modulation of the TGF-β/Smad axis may contribute to both the antifibrotic and anti-invasive effects of Osthole. However, given the dual (pro- and anti-inflammatory) nature of TGF-β signaling, its precise role as a therapeutic target of Osthole likely depends on the specific pathological context and warrants further investigation. Collectively, the ability of Osthole to disrupt TGF-β-driven signaling underscores its therapeutic potential across the inflammation–fibrosis pathological continuum.

Beyond pathway-level regulation, the downstream effects of Osthole on apoptosis, cellular metabolism, and inflammation-related stress responses may also help explain its activity in inflammatory and cancer settings. GO enrichment analysis showed that Osthole-related targets were significantly associated with the regulation of apoptotic signaling, responses to lipopolysaccharide and other bacterial components. Molecular docking further suggested that Osthole may interact with key cell-death executors, including CASP3. Previous studies have shown that Osthole can induce mitochondrial membrane depolarization, disrupt mitochondrial integrity and cellular energy metabolism, and activate the intrinsic apoptotic pathway [108,109]. It is therefore plausible that suppression of pro-survival pathways such as PI3K/Akt and NF-κB may lower the apoptotic threshold of malignant cells or abnormally activated inflammatory cells, thereby facilitating the execution of mitochondrial apoptotic signaling. Osthole has also been reported to promote GSDME/caspase-mediated pore formation in the plasma membrane, leading to membrane rupture and a pyroptosis-like form of cell death. This process may contribute to the removal of damaged cells in inflammatory settings and may also serve as one mechanism for direct tumor-cell elimination [110]. In addition, available evidence suggests that Osthole may disturb cellular metabolic homeostasis and promote bioenergetic stress under certain experimental conditions. For example, Osthole can enhance sodium and calcium channel activity, increase transmembrane ion flux, suppress glycolysis, impair ATP production, inhibit Na^+^/K^+^-ATPase function, and promote ROS accumulation, together driving cell death [111,112,113,114]. This interpretation is broadly consistent with the enrichment results and suggests that upstream signaling changes may converge on downstream effects related to oxidative stress, metabolic imbalance, and cell death. For example, inhibition of the PI3K/Akt/mTOR axis may limit anabolic signaling and energy utilization [115], whereas calcium overload, mitochondrial dysfunction, respiratory-chain impairment, and reduced ATP synthesis may further aggravate cellular bioenergetic stress [53,116].

Nevertheless, these observations should be interpreted cautiously. Although the literature supports apoptosis-related and metabolism-related effects, the current evidence does not justify an overly deterministic formulation of a single terminal effector mechanism operating uniformly across all disease models. A more cautious interpretation is that these downstream events represent recurrent biological consequences observed in multiple contexts. Beyond individual signaling pathways, a recurring theme across the diverse pathological models examined here is that Osthole acts not merely as a suppressor of inflammation, but as a strategic disruptor of disease-sustaining feedback loops. Whether by uncoupling barrier failure from local inflammation in atopic dermatitis, blocking the transition from acute airway inflammation to fibrotic remodeling in asthma, or preventing HMGB1-driven inflammatory amplification in ischemia–reperfusion injury, Osthole consistently intercepts the self-perpetuating cycles that drive disease progression. This system-level perspective may help explain its broad therapeutic potential across seemingly distinct pathological contexts.

Beyond its reported anti-inflammatory and pro-apoptotic effects, our analysis suggests that Osthole may also be relevant to immune-regulatory processes within the tumor microenvironment. Enrichment of the PD-L1 expression and PD-1 checkpoint pathway in cancer raises the possibility that Osthole may intersect with pathways involved in immune evasion. In addition, published evidence indicating inhibition of HMGB1-related inflammatory signaling suggests a possible connection between Osthole-mediated tumor cell death and modulation of local inflammatory cues. However, these observations should be interpreted cautiously. At present, the available evidence is insufficient to conclude that Osthole directly remodels the tumor immune microenvironment, reverses immunosuppression, or converts immune-cold tumors into immune-hot tumors. Instead, our findings offer a mechanistic basis for future studies testing whether Osthole can influence immune-cell infiltration, macrophage polarization, myeloid-derived suppressor cell accumulation, or responsiveness to immune checkpoint blockade in vivo.

Through network pharmacology, molecular docking, molecular dynamics simulations, and literature-based evidence integration, this study established a systematic mechanistic framework for how Osthole may regulate the inflammation–cancer axis. Several limitations should nevertheless be acknowledged. First, network pharmacology analyses depend on existing target-prediction databases and disease-related gene databases. Differences in data sources, prediction algorithms, update frequency, and annotation completeness may introduce database-related bias. Second, the core targets identified through target intersection, PPI network construction, and topological screening should still be regarded as candidate regulatory nodes. Potential false-positive results cannot be excluded, and direct target engagement and functional relevance require further experimental validation. Third, although molecular docking and molecular dynamics simulations provide structural support for potential interactions between Osthole and key targets, these methods are influenced by protein structure selection, binding-pocket definition, scoring functions, and the extent of conformational sampling. In particular, the 100 ns molecular dynamics simulations performed in this study are useful for assessing complex stability within a limited time scale, but they are not sufficient for complete conformational sampling of large proteins. Fourth, this study lacks in vitro and in vivo experimental validation. Therefore, the effects of Osthole on PI3K/Akt, NF-κB, TGF-β/Smad, HMGB1-related signaling, and PD-1/PD-L1-associated immune regulation need to be further confirmed through cell-based assays, animal models, target perturbation experiments, and binding-affinity measurements. Finally, the literature-based evidence integration conducted here was not a systematic review in the strict sense. The disease models discussed in this study were selected as representative and mechanistically relevant examples rather than as a comprehensive representation of the entire inflammation–cancer spectrum. Because the included studies were mainly selected around the computational predictions and representative disease models, some degree of selection bias and confirmation bias may be present. These limitations help define the appropriate scope of our findings: this study provides a systematic candidate-mechanism framework for Osthole intervention in the inflammation–cancer axis and offers prioritized directions for future target validation, mechanistic studies, and translational development. Building on this framework, the further development of Osthole should move toward more systematic translational research. Future studies should combine ADMET modeling with experimental validation to evaluate its absorption, distribution, metabolism, excretion, and potential toxicity, with particular attention to gastrointestinal absorption, blood–brain barrier permeability, CYP450-related metabolic risk, P-gp transport, hepatotoxicity, and cardiotoxicity. Given the poor aqueous solubility and limited oral bioavailability of Osthole [117,118], formulation strategies such as liposomes, polymeric nanoparticles, solid lipid nanoparticles, nanoemulsions, cyclodextrin inclusion complexes, and targeted nanodelivery systems should be explored to improve its solubility, stability, systemic exposure, and delivery efficiency to disease lesions. In addition, its effects on core signaling pathways and tumor immune-related processes should be further evaluated in immunocompetent or humanized animal models. Its potential use in combination with immune checkpoint inhibitors, chemotherapeutic agents, or antifibrotic drugs also warrants further investigation.

## 5. Conclusions

Through an integrative analysis combining network pharmacology, molecular docking, rigorous molecular dynamics simulations, and literature-based evidence synthesis, this study suggests that Osthole may modulate the inflammation–cancer axis through a limited set of shared signaling pathways, particularly PI3K/Akt, NF-κB, and TGF-β/Smad. Crucially, dynamic simulations provided supportive physicochemical evidence, suggesting that Osthole can form stable, energetically favorable complexes with key protein hubs (AKT1, RELA, and TGFB1) within these networks under the simulated conditions. The available evidence also indicates that its pharmacological effects may extend beyond direct anti-inflammatory and cytotoxic actions. The enrichment of PD-1/PD-L1-associated pathways, together with literature-based evidence related to HMGB1 signaling, further suggests a possible connection with immune-regulatory processes and identifies this as a relevant direction for future experimental validation. Although these findings require further in vivo experimental validation, the convergence of system-level predictions and micro-thermodynamic structural evidence supports further investigation of Osthole as a promising multi-target candidate for the coordinated modulation of inflammation-associated tumor progression.

## Figures and Tables

**Table 1:** Enrichment of core targets in key signaling pathways related to the inflammation–cancer axis.

Classification of Mechanisms	Pathway Name (KEGG ID)	p. Adjust
Core signal axis	PI3K-Akt signaling pathway(hsa04151) NF-kappa B signaling pathway(hsa04064)	3.86 × 10^−7^ 3.09 × 10^−6^
Immunity and microenvironment	TNF signaling pathway(hsa04668) IL-17 signaling pathway(hsa04657) PD-L1 expression and PD-1 checkpoint pathway in cancer(hsa05235) Th17 cell differentiation(hsa04659)	2.34 × 10^−10^ 4.24 × 10^−11^ 6.18 × 10^−8^ 1.52 × 10^−7^
Cell fate	Apoptosis(hsa04210)	1.62 × 10^−11^
	Cellular senescence(hsa04218)	8.48 × 10^−7^
Cancer examples	Prostate cancer(hsa05215)	6.25 × 10^−14^
	Hepatocellular carcinoma(hsa05225)	2.25 × 10^−5^
	Non-small cell lung cancer(hsa05223)	5.49 × 10^−4^

**Table 2:** Molecular docking results for Osthole and reference inhibitors across representative targets.

Target	PDB ID	Osthole Binding Energy ΔG (kcal/mol)	Reference Comparator	Comparator Binding Energy ΔG (kcal/mol)	Representative Interacting Residues
AKT1	3O96	−8.3	Ipatasertib	−6.0	ARG-76, GLN-61
BCL2	1G5M	−6.8	HA14-1	−6.6	ASP-196, TYR-9
CASP3	1GFW	−6.2	Isatin	−6.1	SER-251
CASP8	6AGW	−6.7	Z-IETD-FMK	−5.6	ARG-33, GLN-32
CASP9	2AR9	−7.6	Z-LEHD-FMK	−6.6	SER-156
ESR1	2BJ4	−7.6	Tamoxifen	−6.0	ASN-439, GLN-441
HSP90AA1	1BYQ	−7.0	17-AAG	−6.3	ASN-51, THR-184
MAPK3	2ZOQ	−6.9	FR180204	−7.1	GLN-266, LEU-284
MMP9	1ITV	−7.1	Marimastat	−6.4	ARG-173, GLN-178, LEU-176
MTOR	1AUE	−7.9	Rapamycin	−7.8	LYS-2046, VAL-2045
NFKBIA	6Y1J	−7.3	BAY 11-7082	−6.8	ARG-169
PARP1	4OPX	−6.7	3-Aminobenzamide	−6.2	LYS-551, TYR-618
PPARG	2Q59	−8.0	Rosiglitazone	−8.2	LYS-422, SER-428
PTEN	1D5R	−7.3	SF1670	−7.6	ARG-173, TYR-176
PTGS2	5F19	−8.6	Aspirin	−7.1	ALA-202, THR-206, TYR-385
RELA	1BFT	−7.2	Curcumin	−5.5	ARG-238, ASN-239, LYS-240
TGFB1	1KLC	−7.7	LY364947	−6.8	ASN-147, GLN-96, TRP-149

**Table 3:** Representative evidence for the anti-inflammatory and anti-tumor actions of Osthole across inflammatory and cancer disease models.

Disease Model	Representative Mechanisms	Key Biological Outcome	References
Allergic asthma	Promotes airway relaxation via Cav1.2/TRPV1-related mechanisms; suppresses mast-cell activation and allergic inflammation; attenuates TGF-β1-associated epithelial injury, EMT, and airway remodeling.	Reduced airway inflammation, epithelial injury, and remodeling	[35,36,37]
Atopic dermatitis	Suppresses NF-κB-associated inflammation; inhibits Akt phosphorylation and restores ZO-3-related barrier repair; downregulates TLR2-pathway genes and alleviates itch through TRP-related mechanisms.	Improved skin barrier integrity, reduced pruritus, and reduced cutaneous inflammation	[38,39,40]
Rheumatoid arthritis	Suppresses inflammatory signaling and cytokine release, reduces oxidative stress, and inhibits synoviocyte proliferation/migration; may also involve AMPK activation and NLRP3 inhibition.	Reduced synovitis, pannus formation, and joint damage	[41,42]
Renal ischemia–reperfusion injury	Inhibits HMGB1 nuclear release/translocation; suppresses JAK2/STAT3 and NF-κB-related inflammatory signaling; decreases ROS and lipid peroxidation; attenuates oxidative stress-associated tubular apoptosis and mitochondrial dysfunction.	Reduced renal inflammation and tubular injury	[43,44,45,46]
Myocardial fibrosis	Reduces TGF-β1 expression and Smad signaling, limits collagen deposition, and may additionally regulate NF-κB-related and autophagy-associated remodeling.	Attenuated fibrotic remodeling	[47,48,49,50]
Neurodegenerative disease	Suppresses microglial activation in an NRF2-dependent manner, enhances Nrf2/HO-1 signaling, reduces ROS accumulation, and limits neuronal apoptosis and oxidative injury.	Reduced neuroinflammation and neuronal injury	[51,52]
Breast cancer	Induces cell-cycle arrest, mitochondrial dysfunction, calcium imbalance, and ER stress; promotes Bax-associated apoptosis and modulates Akt/ERK/JNK signaling.	Reduced proliferation and enhanced apoptosis in breast cancer cells	[53]
Ovarian cancer	Induces apoptosis and G2/M arrest; suppresses PI3K/Akt-associated survival signaling; engages mitochondrial apoptosis; later evidence suggests ER-mitochondrial stress, autophagy, and pyroptosis-related cell death.	Reduced proliferation and enhanced tumor cell death	[54,55,56]
Lung cancer	Suppresses MMP-9-associated invasion and migration, promotes apoptosis, and perturbs cell-cycle progression, partly through NF-κB- and PI3K/Akt-related signaling.	Reduced invasion/migration and increased apoptosis	[57,58,59]
Hepatocellular carcinoma	Suppresses NF-κB activity, induces apoptosis and G2/M arrest, inhibits proliferation and tumor growth, and may additionally involve AKT/FASN-related metabolism, DNA-damage responses, and GSK-3β/AMPK/mTOR-regulated glycolysis.	Reduced HCC cell growth and enhanced apoptosis	[60,61,62,63,64]
Drug-resistant leukemia	Reverses P-glycoprotein-mediated multidrug resistance; increases intracellular drug accumulation; downregulates MDR1 expression; inhibits PI3K/Akt signaling.	Enhanced chemosensitivity and reduced drug resistance	[65]

The experimental models, doses, routes of administration, and endpoints varied among the cited studies; detailed protocols can be found in the respective references. Despite this heterogeneity, the recurring signaling pathways summarized here highlight the convergent mechanisms that inform the proposed inflammation–cancer continuum.

**Table 4:** Quantitative summary of post-equilibration MD parameters for Osthole-target complexes.

Complex	Equilibrated Phase	Complex RMSD (nm)	Ligand RMSD (nm)	RMSF (nm)	Rg (nm)	SASA (nm^2^)
Osthole-AKT1	25–100 ns	0.396 ± 0.029	0.060 ± 0.017	0.175 ± 0.128	1.417 ± 0.011	81.780 ± 1.663
Osthole-RELA	20–100 ns	0.346 ± 0.025	0.059 ± 0.015	0.162 ± 0.068	2.577 ± 0.016	203.452 ± 3.120
Osthole-TGFB1	10–100 ns	0.208 ± 0.015	0.054 ± 0.018	0.117 ± 0.062	1.930 ± 0.009	147.995 ± 2.287

Values are presented as mean ± SD and were calculated from the equilibrated portion of each 100 ns MD trajectory. RMSF values represent all residues in each simulated complex. The equilibrated intervals were 25–100 ns for Osthole-AKT1, 20–100 ns for Osthole-RELA, and 10–100 ns for Osthole-TGFB1.

## Data Availability

The original contributions presented in the study are included in the article. Further inquiries can be directed to the corresponding author.

## Supplementary Materials

The following supporting information can be downloaded at: https://www.mdpi.com/article/10.3390/cimb48050518/s1.

## Abbreviations

The following abbreviations are used in this manuscript:

MCC	Maximal Clique Centrality
GO	Gene Ontology
KEGG	Kyoto Encyclopedia of Genes and Genomes
P-gp	P-glycoprotein
EMT	Epithelial–mesenchymal Transition
ROS	Reactive Oxygen Species
RA	Rheumatoid Arthritis
IRI	Ischemia–Reperfusion Injury
AD	Atopic Dermatitis
HCC	Hepatocellular Carcinoma
MD	Molecular Dynamics
SASA	Solvent Accessible Surface Area
FEL	Free Energy Landscape
RMSF	Root Mean Square Fluctuation
RMSD	Root Mean Square Deviation
Rg	Radius of gyration
mTOR	mechanistic Target of Rapamycin
PPI	Protein–protein Interaction
PIP_2_	Phosphatidylinositol 4,5-bisphosphate
PIP_3_	Phosphatidylinositol 3,4,5-trisphosphate
DAMPs	Damage-associated Molecular Patterns
MDSCs	Myeloid-derived Suppressor Cells
ZO-3	Zonula Occludens-3
ARE	Antioxidant Response Element
ICIs	ICIs
